# The complete chloroplast genome sequence of the water fern *Ceratopteris thalictroides* (Pteridaceae)

**DOI:** 10.1080/23802359.2022.2135401

**Published:** 2022-11-02

**Authors:** Xing-Feng Liu, Xi-Le Zhou, Yu-Feng Gu, Si-Fan Liu, Jun-Hao Yu, Rui Zhang, Jiang-Ping Shu

**Affiliations:** a Xiangxi Tujia and Miao Autonomous Prefecture Forest Resources Monitoring Center, Jishou, Hunan, China; b Key Laboratory of National Forestry and Grassland Administration for Orchid Conservation and Utilization, Shenzhou, China; c The Orchid Conservation and Research Center of Shenzhen, Guangdong, China; d Xiangxi Tujia and Miao Autonomous Prefecture Institute of Forestry, Jishou, Hunan, China; e Shanghai Center for Plant Stress Biology, National Key Laboratory of Plant Molecular Genetics, Center for Excellence in Molecular Plant Sciences, Chinese Academy of Sciences, Shanghai, China; f Shanghai Chenshan Plant Research Center, Chinese Academy of Sciences, Shanghai, China; g Key Laboratory of Plant Resources Conservation and Sustainable Utilization, South China Botanical Garden, Chinese Academy of Sciences, Guangzhou, Guangdong, China

**Keywords:** *Ceratopteris thalictroides*, Pteridaceae, chloroplast genome, phylogeny

## Abstract

This work determined and analyzed the complete chloroplast genome sequence of *Ceratopteris thalictroides* (Linnaeus) Brongniart 1822 (Pteridaceae). The results indicate that the total chloroplast genome size of *C. thalictroides* is 149,399 bp in length, and the genome contains a large single-copy (LSC) region of 83,580 bp, a small single-copy (SSC) region of 21,241 bp, and a pair of inverted repeat (IR) regions of 22,289 bp. The GC content of *C. thalictroides* is 36.7%. The genome encodes a total of 131 unique genes, including 82 protein-coding genes, 38 tRNA genes, and 8 rRNA genes. The phylogenetic analysis results strongly suggest that *C. thalictroides* is closely related to *C. cornuta*.

The water fern *Ceratopteris thalictroides* (Linnaeus) Brongniart 1822 grows in marshlands and paddy fields and sometimes floats on the water. It was previously classified as a member of Parkeriaceae (Ching 1978) but is now considered a member of Pteridaceae, which is located at the base of the order Polypodiales on the phylogenetic tree (Smith et al. 2006; PPG I 2016; Shen et al. 2018). It is widely distributed worldwide throughout the tropics. Because of the destruction of its habitat and extensive plundering for its edible, ornamental, and medicinal properties, *C. thalictroides* is treated as a second-class protected wild plant in China (National Forestry Administration of the People’s Republic of China & Ministry of Agriculture of the People’s Republic of China 2021). *C. thalictroides* is an excellent model plant because of its short lifespan and diverse mechanisms of reproduction (Li and Wang 1997). To date, no studies on the complete chloroplast genome of *C. thalictroides* have been published. Therefore, we analyzed the complete chloroplast genome of *C. thalictroides* in this study for subsequent molecular phylogenetic analysis.

The *C. thalictroides* sample used in this study was collected from Guantouzui, Hanshou County, Hunan Province, China (111, 58′ 32.0641″ E; 29, 1′ 39.1464″ N; altitude: 9 m); the specimen was deposited at the Chenshan Herbarium (CSH, Shanghai, China) and assigned voucher number Fern09721, Zhou XL, 2018 (Zhang Rui, 249967429@qq.com).

The genomic DNA was extracted from the silica gel dried leaf of *C. thalictroides* and sequenced on the Illumina HiSeq platform (Shanghai Majorbio Bio-pharm Technology Co., Ltd., Shanghai, China). The plastid genome was assembled using GetOrganelle (Jin et al. 2018) with the chloroplast genome of *C. cornuta* (accession number: MH173068) as the reference sequence. The assembled chloroplast genome was annotated by Geneious Prime (Biomatters Ltd., Auckland, New Zealand) (Kearse et al. 2012). Finally, the complete chloroplast genome of *C. thalictroides* was obtained; the genome sequence data are openly available in the GenBank database of the National Center for Biotechnology Information (NCBI) at https://www.ncbi.nlm.nih.gov/ under accession number OK524221.

The results indicate that the chloroplast genome size of *C. thalictroides* (OK524221) is 149,399 bp in length, and the genome contains a large single-copy (LSC) region of 83,580 bp, a small single-copy (SSC) region of 21,241 bp, and a pair of inverted repeat (IR) regions of 22,289 bp. The GC content of *C. thalictroides* is 36.7%. The genome encodes a total of 131 unique genes, including 82 protein-coding genes, 38 tRNA genes, and 8 rRNA genes.

For the molecular phylogenetic analysis, the complete chloroplast genomes of 15 ferns were downloaded from GenBank to obtain a phylogenetic tree containing *C. thalictroides*. The sequences were aligned by MAFFT (Katoh and Standley 2013). The phylogenetic tree was constructed using the IQ-TREE (maximum likelihood) method in PhyloSuite, and branch supports were constructed with ultrafast bootstrap approximation (Guindon et al. 2010; Minh et al. 2013; Nguyen et al. 2015; Zhang et al. 2020). The results revealed that *C. thalictroides* is a sister of *C. cornuta* MH173068 (Figure 1). This is consistent with previous studies on the Pteridaceae phylogenetic tree.

**Figure 1.**
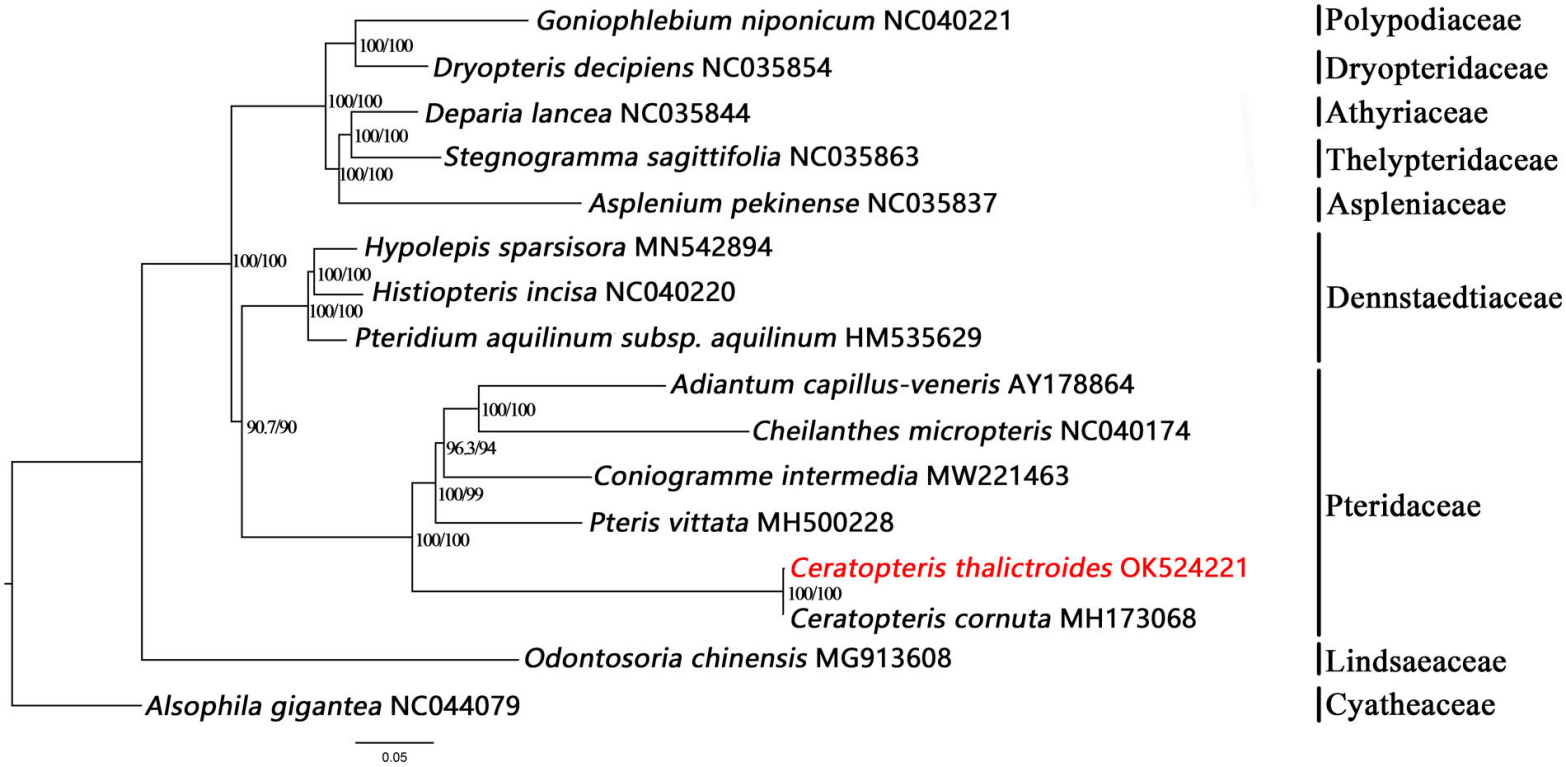
Maximum-likelihood phylogenetic tree based on 16 complete chloroplast genome sequences. Bootstrap support is indicated for each branch.

## Data Availability

The genome sequence data that support the findings of this study are openly available in the GenBank database of NCBI at https://www.ncbi.nlm.nih.gov (https://www.ncbi.nlm.nih.gov/nuccore/OK524221.1/) under accession no. OK524221. The associated BioProject, Sequence Read Archive, and BioSample numbers are PRJNA733526, SRR14683085, and SAMN19414834, respectively.

## Plant material collection statement

The species is widely distributed in China; the plant materials were not obtained from nature reserves, and the chloroplast genome sequencing research did not affect the population, so collection licenses were not needed.
